# CASE REPORT Febrile Ulceronecrotic Mucha-Habermann Disease

**Published:** 2010-07-16

**Authors:** Patrick S. Harenberg, Manuel Hrabowski, Henning Ryssel, Emre Gazyakan, Günter Germann, Holger Engel, Matthias A. Reichenberger

**Affiliations:** Department of Hand, Plastic, and Reconstructive Surgery Burn Center; BG Trauma Center Ludwigshafen, Department of Plastic and Hand Surgery, University of Heidelberg, Ludwig-Guttmann, Ludwigshafen, Germany

## Abstract

**Objective:** Case report and review of the current literature about febrile ulceronecrotic Mucha-Habermann disease (FUMHD). **Methods:** Review of our patient's medical records and of the current literature. **Results:** The FUMHD is a rare and potentially lethal type of pityriasis lichenoides et varioliformis acuta. It is characterized by the sudden onset of ulceronecrotic skin lesions associated with high fever and systemic symptoms. Because of a high case-fatality rate it requires quick and decisive action. Only 40 cases of this severe form of the disease have been reported in the literature to date. We present the case of a 30-year-old male patient with severe FUMHD who was successfully treated in our burn intensive care unit after failed treatment at a dermatological hospital. The patient was treated with topical antiseptics, moisturizers, and artificial skin substitutes, as well as systemic immunosuppressive therapy (glucocorticoids) with which we were able to control the disease activity so that healing of the patient's skin lesions could be achieved. **Conclusion:** Patients with FUMHD should be treated in a specialized center for severely burned patients. Only such centers can provide the structural and logistical capacities necessary for the treatment of such extensive superficial wounds.

Febrile ulceronecrotic Mucha-Habermann disease (FUMHD) is a rare and potentially lethal type of pityriasis lichenoides et varioliformis acuta (PLEVA). Characteristics of FUMHD include a fulminant course of the disease, painful ulceronecrotic erosions, fever, and severe systemic manifestations. In current literature only 40 cases with an overall case fatality rate of 20% are described. Until today the knowledge about the etiology of this disease is only fragmentary, but an association with viral and lymphoproliferative diseases is being discussed.

We are presenting the case of a 30-year-old man who was successfully treated for a severe case of FUMHD with an affected body surface area of 65%. The treatment took place in our burn intensive care unit.

## CASE REPORT

Eleven months after successful treatment for Hodgkin disease in stage IIIA (chemotherapy, BEACOPP regimen), the patient developed a case of PLEVA/FUMHD. He initially received dermatological treatment with topical and systemic agents (cefuroximaxetil, methothrexate, prednisolone, aciclovir, moxifloxacin, and doxorubicin). Despite this treatment new skin lesions appeared and the patient was admitted to our burn intensive care unit. On admission, the patient's whole integument (with an emphasis on the upper body) was covered with sharply bounded erythematous papules and plaques. Solitary necroses were visible on the patient's abdomen and back. In addition, the patient developed disseminated ulceronecrotic lesions in the course of his disease.

**Figure 1:**
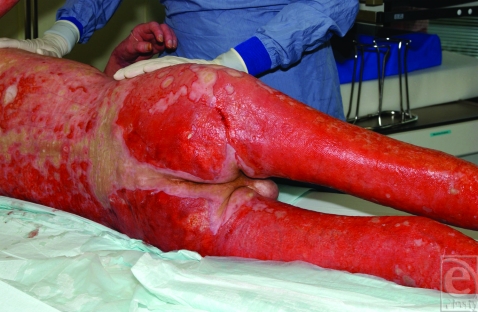
Skin lesions on admission to the burn ICU.

On admission, the onset of sepsis and colonization of the skin lesions with methicillin-resistant *Staphylococcus aureus* and *Pseudomonas* were the additional factors complicating this case. The physical examination showed painful, disseminated, erythematous, and papulosquamous lesions, partially with hemorrhagic ulcerations (Fig 1).

Initially we conducted topical treatment with Vaseline gauze and a polyhexanide solution (Lavasept) as well as systemic treatment with 1 mg/kg/d prednisolone. Balanced fluid substitution was initiated after insertion of a central catheter.

Because microbiological tests (smears) of the patient's skin lesions revealed a superinfection with *Staphylococcus aureus*, *Pseudomonas aeruginosa*, and enterobacteriaceae a calculated systemic antibiotic therapy was initiated. Dressings were changed every second day. Topical treatment was continued with Suprathel as well as Aquacell. This therapy regimen was supplemented by dietary and physical therapy as well as psychotherapeutic attendance.

**Figure 2:**
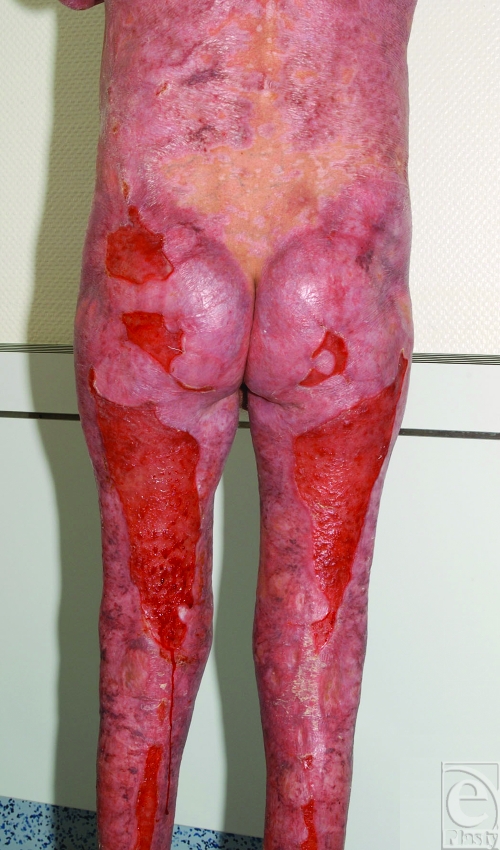
Result shortly before discharge from our hospital (5.5 months later).

With this treatment we were able to control the disease and the skin lesions were healed (Fig 2).

## DISCUSSION

The FUMHD is a rare and potentially lethal type of PLEVA. Characteristics of FUMHD include a fulminant course of the disease, painful ulceronecrotic eruptions, fever, and severe systemic manifestations.

The FUMHD was first described by Dagos in 1966 and until today there are only 40 cases documented in international literature.^1^ According to the reports, the majority of cases are children and young adults (predominantly males, mean age 27.4 years, range 4-82 years). Literature describes the disease's case-fatality rate at about 20%, with the rate being 0% in children and up to 33.3% in adults.^2^

Until today the knowledge about the etiology of this disease is only fragmentary, but an association with viral diseases is being discussed. In these cases an immediate correlation between management of the underlying disease and prognosis of the FUMHD is described.^3^,^4^

In the early course of the disease differential diagnoses may include chickenpox, Gianotti-Crosti syndrome, lichen planus, pityriasis rosea, and guttate psoriasis. The FUMHD either develops spontaneously (as was the case in our patient) or in preexisting pityriasis lichenoides. There is proof that pityriasis lichenoides and PLEVA as well as lymphomatoid papulomatosis might be a part of the spectrum of benign clonal T-cell proliferative disorders.^5^^-^7

Symptoms include feelings of severe illness, fever, and painful erythematous and papulosquamous eruptions that gradually shift to hemorrhagic ulcerations. Systemic manifestations include diarrhea, abdominal pain, pneumonia, splenomegaly, arthritis, sepsis, or megaloblastic anemia.^1^ In the majority of patients, blood tests show leukocytosis, anemia, elevated C-reactive protein, and elevated liver enzymes. An association of FUMHD with elevated blood levels of TNF-α as been described.^8^

The FUMHD is a great challenge for the treating physicians. Literature recommends immunosuppressive monotherapy with methothrexate or cyclosporine or a combination of one of these with high-dose glucocorticoids. Some authors also described partial success in the treatment of FUMHD with high-dose immunosuppressive therapy in combination with virostatic drugs and antibiotics.^9^,^10^ A preemptive antibiotic therapy for prevention of infections should always be administered. The extensive loss of skin and soft tissue in combination with a quick deterioration of the patient's general condition is similar to the condition of patients with severe burns. Thus, patients with FUMHD should receive the same supportive intensive care as the burn victims.

As in the treatment of burn victims we utilized balanced fluid substitution, regular check of inflammatory parameters, antiseptic topical treatment (Lavasept), nonadhesive wound dressings (Vaseline gauze), and dietary and physical therapy as well as psychotherapeutic attendance.

## CONCLUSION

The FUMHD is characterized by a high case-fatality rate and requires quick and decisive action. A search for a focus of a viral or lymphoproliferative disease as well as an early high-dose immunosuppressive, antibiotic, and topical therapy in a specialized intensive care unit should have highest priority.

These conditions as well as the existing structural and logistical possibilities that a center for severely burned patients offers for the treatment of large wounds predestine patients with FUMHD for treatment in such a center.
